# Recommendations for reporting of systematic reviews and meta-analyses of diagnostic test accuracy: a systematic review

**DOI:** 10.1186/s13643-017-0590-8

**Published:** 2017-10-10

**Authors:** Trevor A. McGrath, Mostafa Alabousi, Becky Skidmore, Daniël A. Korevaar, Patrick M. M. Bossuyt, David Moher, Brett Thombs, Matthew D. F. McInnes

**Affiliations:** 1 Faculty of Medicine, University of Ottawa, Ottawa, ON Canada; 2 Department of Radiology, McMaster University, Hamilton, ON Canada; 3 Ottawa Hospital Research Institute, Ottawa, ON Canada; 4 Department of Clinical Epidemiology, Biostatistics and Bioinformatics, Academic Medical Center, University of Amsterdam, Amsterdam, the Netherlands; 5 Clinical Epidemiology Program, Ottawa Hospital Research Institute, Ottawa, ON Canada; 6 Lady Davis Institute of the Jewish General Hospital and Department of Psychiatry, McGill University, Montreal, Quebec Canada; 7 University of Ottawa Department of Radiology, Clinical Epidemiology Program, Ottawa Hospital Research Institute, Room c159 Ottawa Hospital Civic Campus, 1053 Carling Ave, Ottawa, ON K1Y 4E9 Canada

## Abstract

**Background:**

This study is to perform a systematic review of existing guidance on quality of reporting and methodology for systematic reviews of diagnostic test accuracy (DTA) in order to compile a list of potential items that might be included in a reporting guideline for such reviews: Preferred Reporting Items for Systematic Reviews and Meta-Analyses of Diagnostic Test Accuracy (PRISMA-DTA).

**Methods:**

Study protocol published on EQUATOR website. Articles in full text or abstract form that reported on any aspect of reporting systematic reviews of diagnostic test accuracy were eligible for inclusion. We used the Ovid platform to search Ovid MEDLINE®, Ovid MEDLINE® In-Process & Other Non-Indexed Citations and Embase Classic+Embase through May 5, 2016. The Cochrane Methodology Register in the Cochrane Library (Wiley version) was also searched. Title and abstract screening followed by full-text screening of all search results was performed independently by two investigators. Guideline organization websites, published guidance statements, and the Cochrane Handbook for Diagnostic Test Accuracy were also searched. Preferred Reporting Items for Systematic Reviews and Meta-Analyses (PRISMA) and Standards for Reporting Diagnostic Accuracy (STARD) were assessed independently by two investigators for relevant items.

**Results:**

The literature searched yielded 6967 results; 386 were included after title and abstract screening and 203 after full-text screening. After reviewing the existing literature and guidance documents, a preliminary list of 64 items was compiled into the following categories: title (three items); introduction (two items); methods (35 items); results (13 items); discussion (nine items), and disclosure (two items).

**Conclusion:**

Items on the methods and reporting of DTA systematic reviews in the present systematic review will provide a basis for generating a PRISMA extension for DTA systematic reviews.

## Background

In their 2015 report titled “Improving Diagnosis in Healthcare”, the National Academy of Medicine identified a better understanding of the performance of diagnostic tests as an imminent priority for patient safety [1]. Systematic reviews, which incorporate findings from multiple primary studies, can increase confidence in our understanding of the accuracy of diagnostic tests in detecting medical conditions or diseases [2]. Systematic reviews and meta-analyses are cited more than any other study design and are prioritized in clinical practice guidelines [3–5]. Consistent with this, the number of systematic reviews, including those on diagnostic test accuracy (DTA), has grown extremely rapidly over the past decade [6, 7].

When systematic reviews and meta-analyses are poorly reported, readers are not able to assess the quality of the review and its underlying primary studies or to weigh the applicability of its conclusions. Thus, incomplete or inaccurate reports that do not transparently and completely convey review methods and results may mislead readers, rather than clarify the true value of a test. This contributes to waste of scarce medical research resources [8, 9] and hinders efforts to ensure the reproducibility of research. Previous studies have shown that many published DTA systematic reviews are not adequately reported [10, 11].

The Preferred Reporting Items for Systematic Reviews and Meta-Analyses (PRISMA) statement is a 27-item checklist and flow diagram that aims to provide guidance on complete and transparent reporting of systematic reviews [12]. Use of reporting guidelines, such as PRISMA, is associated with more informative reporting of medical research [10]. PRISMA was developed primarily for systematic reviews of medical interventions. While DTA systematic reviews share some common elements with intervention reviews, there are important differences. Thus, some items in the original PRISMA checklist may not apply to DTA reviews, and some essential items necessary for reporting DTA systematic reviews may be lacking [2, 6, 13, 14]. Existing guidance for reporting of DTA systematic reviews is limited to non-systematic “expert opinion” [2, 15, 16], guidance on specific methodologic items [6, 17], or work that is not yet complete [18].

The PRISMA-DTA group is developing an extension for DTA systematic reviews and meta-analyses. As the initial step, we performed a systematic review of existing guidance on reporting of DTA systematic reviews in order to compile a list of potential items that might be included in a reporting guideline for such reviews, the PRISMA extension for DTA (PRISMA-DTA).

## Methods

The protocol for this review is available on the EQUATOR network’s website (http://www.equator-network.org/) in “guidelines under development” [19].

### Database search

To identify published articles pertaining to reporting of DTA systematic reviews, an experienced medical information specialist (BS) developed a search strategy through an iterative process in consultation with the review team. The strategy was peer-reviewed prior to execution by another senior information specialist using the PRESS checklist [20]. Using the Ovid platform, we searched Ovid MEDLINE® and Ovid MEDLINE® In-Process & Other Non-Indexed Citations and Embase Classic+Embase on May 5, 2016. We also searched the Cochrane Methodology Register in the Cochrane Library, which contains records published July 2012 and earlier, (Wiley version) on the same date. Strategies used a combination of controlled vocabulary (e.g., “Diagnostic Tests, Routine,” “Review Literature as Topic,” “Publication Bias”) and keywords (e.g., “DTA,” “systematic review,” “reporting”). Vocabulary and syntax were adjusted across databases. There were no date or language restrictions on any of the searches. Specific details regarding search strategies appear in Appendix 1.

### Inclusion/exclusion criteria, study selection, and data extraction

We included articles in full-text or abstract form that reported on any aspect of reporting DTA systematic reviews. Specifically, we included studies that evaluated the quality of reporting of any aspect of DTA systematic reviews and studies that provided guidance or suggestions as to how a DTA systematic review should be performed.

Titles and abstracts of all search results were screened independently for potential relevance by two investigators (MA, MDFM). For any citation deemed potentially relevant, full texts were retrieved and independently assessed in duplicate for inclusion with disagreements being resolved by consensus (TAM, MDFM). To facilitate the extraction process, studies were divided into several categories pertaining to the specific reporting topics: assessment of quality of reporting, general guidance on performing or reporting DTA systematic reviews, guidance on search methods for primary DTA studies, assessment of heterogeneity, pooling and meta-analysis methods, assessment of publication bias, risk of bias, and “other.” Reference list of included sources is provided in Appendix 2.

In addition to sources related to DTA systematic reviews, the following sources were reviewed: reporting guideline organizations’ websites (Enhancing the QUAlity and Transparency of Health Research (EQUATOR) [21]), guidance for reporting systematic reviews and meta-analyses of other types of research (Meta-analysis of Observational Studies in Epidemiology (MOOSE) [22], PRISMA [12], PRISMA extensions [23–27]), guidance for reporting diagnostic test accuracy studies (STARD 2015 [28], STARD for abstracts), guidance for, or tools for assessing the methodologic quality of systematic reviews and meta-analyses (A Measurement Tool to Assess Systematic reviews (AMSTAR) [29], risk of bias in systematic reviews (ROBIS) [30], Methodological Expectations of Cochrane Intervention Reviews (MECIR) [31]), and The Cochrane Handbook for Systematic Reviews of Diagnostic Test Accuracy (completed chapters) [18]. Post hoc assessment of the following items not included in the initial search was done: the Agency for Healthcare Research and Quality (AHRQ) Methods Guide for Comparative Effectiveness Research, the Institute of Medicine’s 2011 Standards for Systematic Reviews and the Centre for Reviews and Dissemination guidance [32–34]. No additional items were generated from these sources.

The PRISMA and STARD 2015 checklists were initially assessed independently and in duplicate in order to compile a list of potentially relevant items for the PRISMA-DTA statement. Any item that was deemed possibly relevant to DTA systematic reviews by either investigator was included. Next, all other guidance documents (reporting checklists, The Cochrane Handbook for Systematic Reviews of Diagnostic Test Accuracy, etc.) and full texts of potentially relevant records were similarly assessed in duplicate for additional potentially relevant items (TAM, MDFM). Again, any item that was deemed possibly relevant to DTA systematic reviews by either investigator was included. Items deemed relevant may have had wording changed from the original source to make them more applicable to systematic reviews of diagnostic test accuracy and/or broken into multiple sub-items to facilitate the Delphi process for PRISMA-DTA. All included items were used to generate a comprehensive summary of existing guidance on reporting of DTA systematic reviews.

## Results

### Database search

**Fig. 1:**
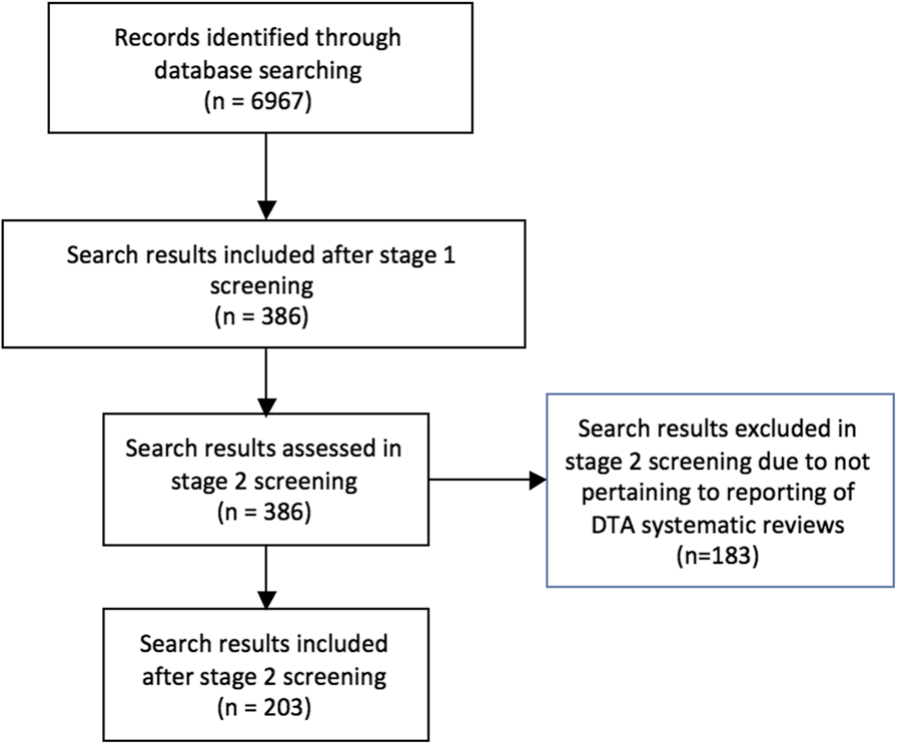
Study flow diagram

The database search yielded 6967 results. After title and abstract screening, 386 results remained. This was further reduced to 203 results after full-text screening (Fig. 1 **)**.

### Identification of potentially relevant items

After searching the existing literature and guidance documents, a preliminary list of 64 unique items was compiled and divided into the following categories mirroring the PRISMA statement: title (three items); introduction (two items); methods (35 items); results (13 items); discussion (nine items), and disclosure (two items). The methods section was further divided into eligibility criteria and search strategy (10 items), study selection and data extraction (seven items), primary study data items that should be provided (one item containing 10 sub-items.), risk of bias and heterogeneity (six items), and summary measures and statistics (11 items). The identified items along with citations for the sources from which they were taken are presented in Table 1; shaded items on the table indicate items specific to diagnostic accuracy systematic reviews, while unshaded items represent more general guidance for systematic reviews.

**Table 1:** Potential relevant items for PRISMA-DTA checklist. Items deemed by the authors to apply specifically to DTA reviews are in **Bold**

	Item	Ref
	Title	
1	Identify the report as a systematic review, meta-analysis or both	[12]
**2**	**Identify the report as a study of diagnostic accuracy using at least one measure of accuracy**	[28]
**3**	**State whether the report is a comparative (one diagnostic test vs. another) or a non-comparative review**	[37, 38]
	Introduction	
**4**	**State the scientific and clinical background, including the intended use and clinical role of the index test (e.g., triage test, add-on test, or replacement test**	[39]
5	List review objective using PICO format (participant characteristics, intervention, comparison, outcome)	[12]
	Methods: protocol eligibility, and search	
6	Indicate if a review protocol exists, where it can be accessed and, if available, registration number	[12]
7	Report deviations from the original protocol	[31]
8	Report which outcomes are considered primary and secondary	[31]
9	Describe all information sources and the date of search	[12]
10	Report restrictions to search strategy (language, publication status, dates)	[31]
11	Present full electronic search strategy for at least one database, including any limits used, such that it could be repeated	[12]
12	Report whether hand searching of reference lists was done	[31]
13	Describe methods to ensure that overlapping patient populations were identified and accounted for	[31]
14	List any search of the gray literature including search of study registries	[31]
15	Specify criteria for eligibility	[12]
	Methods: study selection and data collection	
16	Report the process for selecting studies (i.e., screening, full-text eligibility)	[12]
17	Provide an appendix with studies excluded, with reasons for exclusion, during full-text screening	[12]
18	Describe method of data extraction from reports	[12]
19	Report which data items were extracted from included studies	[12]
20	Report how studies for which only a subgroup of participants is relevant to the review will be handled	[31]
**21**	**Report how “indeterminate” or “missing” results for either the index test or reference standard were dealt with in the analysis**	[40]
22	Report if and how any parameters beyond test accuracy will be evaluated (e.g., cost-effectiveness, mortality)	[46]
	Methods: primary study data items	
**23**	**(a) Patient demographic information (age, gender)**	[2, 12, 28]
	**(b) Target condition definition**	
	**(c) Index test**	
	**(d) Reference standard**	
	**(e) Positivity thresholds**	
	**(f) Blinding information**	
	**(g) Clinical setting**	
	**(h) Disease prevalence**	
	**(i) Cross-tabulation of index test with reference standard (2 × 2 table)**	
	**(j) Funding sources**	
	Methods: risk of bias and heterogeneity	
**24**	**Report how included individual studies will be assessed for methodological quality (e.g., QUADAS-2)**	[14]
25	Describe if and how “piloting” the risk of bias tool was done	[14]
26	List criteria used for risk of bias ratings applied during the review	[31]
27	Describe methods for study quality assessment	[12]
**28**	**Provide measures of consistency** (e.g., tau^2^) for each meta-analysis	[12]
29	Describe test used to assess for publication bias	[12]
	Methods: summary measures and statistics	
**30**	**State the principal summary measures of diagnostic accuracy to be assessed**	[28]
**31**	**Report whether summary measures were calculated on a per-patient or per-lesion basis**	[31]
**32**	**Report pre-defined criteria for minimally acceptable test performance**	[42]
**33**	**State how multiple readers of an index test were accounted for**	[17]
**34**	**Report the statistical method used for meta-analysis (e.g., hierarchical model)**	[2]
**35**	**State which software package and macros was used for meta-analysis**	[6]
**36**	**Report any programming deviations made from published software packages**	[6]
**37**	**If comparative design, state the statistical methods used to compare test accuracy**	[28]
38	Describe methods of additional analyses (e.g., subgroup), indicating whether pre-specified	[12]
39	Report how subgroup analyses were performed	[31]
40	When performing meta-regression report the form of factors being explored (categorical vs. continuous) and the cut-off points used	[41]
	Results	
41	Report studies from screen to inclusion, ideally with a flow diagram	[12]
42	For each study, present characteristics for which data were extracted and provide the citations	[12]
**43**	**Present data on risk of bias of each study on a per-item or per-domain basis**	[12, 14, 35]
44	Present results of any assessment of publication bias	[12]
45	Report any adverse events or harms from index test or reference standard	[31]
**46**	**For each study report 2 × 2 data (TP, FN, FP, TN)**	[43, 45]
**47**	**For each study report summary estimates of accuracy and confidence intervals**	[28]
48	Report each meta-analysis including confidence intervals and measures of consistency (e.g., tau2)	[12]
**49**	**Graphically display results with an ROC curve or forest plots of sensitivity and specificity**	[44]
50	Report additional analyses (e.g., meta-regression)	[12]
51	Report risk of bias in the synthesis (e.g., analyses stratified by risk of bias)	[31]
52	Report summary of findings table with main outcomes and issues re: applicability of results	[31]
53	Report “frequency” tables of 2 × 2 data demonstrating potential findings in a patient population based on the prevalence	[45]
	Discussion	
54	Summarize findings including implications for practice	[12, 28]
55	Provide a general interpretation of the results in the context of other evidence and implications for future research	[12]
56	For comparative design, report whether conclusions were based on direct vs. indirect comparisons	[37]
57	Discuss the implications of any missing data	[31]
58	Discuss applicability concerns to different populations/settings	[14, 45]
59	Discuss quality of included studies when forming conclusions	[36]
60	Account for any statistical heterogeneity when interpreting the results	[31]
61	Discuss the potential impact of reporting biases	[31]
62	Discuss the five GRADE considerations (study limitations, consistency of effect, imprecision, indirectness, and publication bias)	[31]
	Disclosure	
63	Describe sources of funding for the review and role of funders	[12]
64	Report potential relevant conflicts of interest for review investigators	[36]

“Ref” = source reference(s) for the item

Items were taken from 19 unique sources with publication dates between 2007 and 2016, a combination of guidance documents and some of the 203 search results. The 19 sources included the PRISMA statement [12], the PRISMA Explanation and Elaboration document [35], STARD 2015 [28], MECIR [31], AMSTAR [36], QUADAS-2 [14], eight research articles [6, 17, 37–42], two reviews [2, 43], two DTA statistical methodology overviews [44, 45], and one conference abstract [46]. Many of the 203 included results contained redundant information; one source was cited per item.

### Summary of rationale for relevant items

This section will highlight some of the items that are proposed that have particular relevance to DTA systematic reviews.

*Title*: The potential items listed in this section aim to clearly identify “big picture” components of study design; this not only allows immediate reader comprehension, but enhances indexing and searchability. Items 1 and 2 are drawn from PRISMA and STARD 2015 and require that the title indicate that the study is a systematic review (item 1) and is a study of diagnostic accuracy (item 2). Item 3 required reporting on whether the study design is comparative (one test vs. another) or non-comparative; comparative design is increasingly important, common, and associated with methodologic challenges [37].

*Introduction*: Item 4 requires framing the role of the index test in the existing clinical pathway; understanding the clinical role of a test is essential to generalizability of findings. For example, if a test evaluation focuses on a “triage” test (e.g., d-dimer for determination of pre-test probability prior to CT pulmonary angiogram), it may not be appropriate to generalize its use as a “replacement” test (e.g., d-dimer as a *replacement* for CT). The performance of diagnostic tests is variable depending on the specific clinical scenario [28, 47].

*Methods—protocol*, *eligibility*, *and search*: All items in this section are generalizable to all systematic reviews; none were deemed to be specific to DTA systematic reviews.

*Methods*—*study selection and data collection*: Multiple items in this section focus on specific details of the search strategy and are aimed at enhancing reproducibility. None of these is of particular specific relevance to DTA reviews; however, detail additional to that recommended by PRISMA has been listed since subsequent systematic review methodologic recommendations have suggested their inclusion [31].

*Methods—primary study data items*: Item 25 focuses on which characteristics from primary studies included in a review should be reported. Several aspects of this item are unique to DTA systematic reviews, such as index test, reference standard, target condition definition, test positivity thresholds, and clinical setting. All this information is vital for readers to make an appropriate assessment of the review.

*Methods—risk of bias and heterogeneity*: Assessment of study quality and heterogeneity are not unique to DTA reviews. However, study quality assessment for diagnostic accuracy studies includes assessment of risk of bias and concerns regarding applicability, thus the quality assessment tool used in DTA reviews should capture and report these issues (item 24) [14]. Additionally, since sensitivity and specificity are correlated, univariate measures of heterogeneity, such as *I* ^2^, are typically not appropriate to report heterogeneity in diagnostic test accuracy reviews. Thus, heterogeneity may be reported either qualitatively or using measures that account for the correlation between sensitivity and specificity (item 28) [2].

*Methods—summary statistics*: Multiple readers may interpret an index test. How this is accounted for statistically may affect the results and, therefore, should be reported (item 33) [17]. An important difference in DTA meta-analysis from interventions is the correlation between sensitivity and specificity. Thus, it is very important to report the statistical model used for meta-analysis so readers can determine the impact of these methods on the results (item 34) [6].

*Results*: In order to facilitate reproduction of analyses and to make it clear to the readers which data was meta-analyzed, 2 × 2 data for each study included in meta-analyses should be made available (item 46) [43, 45].

*Discussion and disclosure*: All items in this section are generalizable to all systematic reviews; none was deemed to be specific to DTA systematic reviews.

## Discussion

We consulted existing guidance on the reporting of systematic reviews and the published literature related to the conduct and reporting of DTA systematic reviews to identify 64 potential items for reporting DTA systematic reviews. The systematic, comprehensive search categorized by manuscript section builds on prior work, which has been based on non-systematic searches and expert opinion. The items identified will form the basis of a Delphi process that will be conducted to generate the PRISMA-DTA checklist. Items have been broken down into single concepts or descriptors for the Delphi process. During the Delphi process, suggestions from the PRISMA-DTA group will be incorporated. Thus, some items may not appear on the final PRISMA-DTA checklist. Additionally, PRISMA-DTA group members may propose additional items during the Delphi process. Wording of items as presented here may also be adjusted at the PRISMA-DTA consensus meeting. Therefore, it is advised to consult the final checklist after it has been published for use in guiding reporting systematic reviews of diagnostic test accuracy.

This evaluation improves on prior work, which has largely been based on non-systematic reviews, and expert opinion. The work is a small but essential step towards a clear reporting guideline for DTA systematic reviews. Future work should not only include creating the PRISMA-DTA checklist, but evaluating for “baseline” adherence to PRISMA-DTA in order to guide knowledge translation interventions aimed at targeted improvements for reporting of DTA systematic reviews.

### Strengths and limitations

This systematic review benefits from a comprehensive, expert, peer-reviewed search, duplicate extraction, and categorization of potentially relevant items by manuscript section which mirrors the format of the PRISMA checklist. Limitations of our systematic review are that we did not formally assess the quality of sources for included items, we provide only a qualitative summary, and we may not have identified potentially relevant items from work yet to be published. We believe that many of these shortcomings will be addressed in the process for generation of the PRISMA-DTA checklist as outlined in our complete study protocol [48].

## Conclusions

The reporting of DTA systematic reviews is often incomplete [10, 11, 49]. Incomplete reporting has been identified as a preventable source of waste in biomedical research [43]. Therefore, a reporting guideline specific to DTA systematic reviews is needed to reduce waste, increase utility, and facilitate reproducibility of these reviews. This systematic review is the first step towards gathering all relevant evidence pertinent to reporting of DTA systematic reviews. This step is critical in the EQUATOR network’s established guidance for reporting guidelines development [50]. This information will serve as the substrate for a PRISMA-DTA extension to guide reporting of DTA systematic reviews and will complement the more than 300 reporting guidelines indexed by the EQUATOR Network [21].

## Appendix 1

Search Strategy 2016 May 5 Ovid Multifile Database: Embase Classic+Embase <1947 to 2016 May 04>, Ovid MEDLINE(R) In-Process & Other Non-Indexed Citations and Ovid MEDLINE(R) <1946 to Present>

Search Strategy:

---------------------------

1 “Diagnostic Techniques and Procedures”/ (80885)

2 exp Diagnostic Imaging/ (2015520)

3 “Diagnostic Tests, Routine”/ (73316)

4 (diagnos* adj3 test*).tw,kw. (153714)

5 (diagnos* adj3 accura*).tw,kw. (155998)

6 (test* adj3 accura*).tw,kw. (29472)

7 (diagnos* adj3 compar*).tw,kw. (53752)

8 diagnostic stud$3.tw,kw. (12147)

9 DTA.tw,kw. (5191)

10 (DTAR or DTARs).tw,kw. (34)

11 or/1-10 (2464773)

12 meta analysis.pt. (65207)

13 meta-analysis as topic/ (26801)

14 (meta-analy* or metanaly* or metaanaly* or met analy* or integrative research or integrative review* or integrative overview* or research integration or research overview* or collaborative review*).tw. (214034)

15 (systematic review* or systematic overview* or evidence-based review* or evidence-based overview* or (evidence adj3 (review* or overview*)) or meta-review* or meta-overview* or meta-synthes* or “review of reviews” or technology assessment* or HTA or HTAs).tw. (251988)

16 exp Technology assessment, biomedical/ (21442)

17 “Review Literature as Topic”/ (56641)

18 or/12-17 (492218)

19 11 and 18 (22011)

20 exp Quality Control/ (351525)

21 Publishing/ (61930)

22 Publication Bias/ (37246)

23 Research Report/ (33327)

24 Periodicals as Topic/ (158426)

25 Checklist/ (15858)

26 Research Design/ (1691506)

27 exp Reproducibility of Results/ (483550)

28 ((report* or method* or publicat*) adj3 (assess or apprais* or bias* or characteristic* or criteri* or critiqu* or evaluat* or guidance* or guideline* or quality or checklist* or check list* or recommend* or score$1 or scoring or standard*)).tw,kw. (665469)

29 reporting.tw,kw. (275122)

30 methodolog*.ti,kw. (76262)

31 PRISMA.tw,kw. (5494)

32 or/20-31 (3475491)

33 19 and 32 (6906)

34 33 use prmz (3684)

35 exp. diagnostic test/ (840676)

36 (diagnos* adj3 test*).tw,kw. (153714)

37 diagnostic accuracy/ (200586)

38 (diagnos* adj3 accura*).tw,kw. (155998)

39 (diagnos* adj3 compar*).tw,kw. (53752)

40 diagnostic test accuracy study/ (44252)

41 (test* adj3 accura*).tw,kw. (29472)

42 DTA.tw,kw. (5191)

43 (DTAR or DTARs).tw,kw. (34)

44 diagnostic stud$3.tw,kw. (12147)

45 or/35-44 (1319550)

46 meta-analysis/ (173526)

47 “systematic review”/ (105948)

48 “meta analysis (topic)”/ (26341)

49 (meta-analy* or metanaly* or metaanaly* or met analy* or integrative research or integrative review* or integrative overview* or research integration or research overview* or collaborative review*).tw. (214034)

50 (systematic review* or systematic overview* or evidence-based review* or evidence-based overview* or (evidence adj3 (review* or overview*)) or meta-review* or meta-overview* or meta-synthes* or “review of reviews” or technology assessment* or HTA or HTAs).tw. (251988)

51 or/46-50 (475344)

52 45 and 51 (20896)

53 medical literature/ (137121)

54 quality control/ (184009)

55 publishing/ (61930)

56 publication/ (145797)

57 checklist/ (15858)

58 reproducibility/ (170497)

59 ((report* or method* or publicat*) adj3 (assess or apprais* or bias* or characteristic* or criteri* or critiqu* or evaluat* or guidance* or guideline* or quality or recommend* or checklist* or check list* or score$1 or scoring or standard*)).tw,kw. (665469)

60 reporting.tw,kw. (275122)

61 methodology/ (1646967)

62 methodolog*.ti,kw. (76262)

63 PRISMA.tw,kw. (5494)

64 or/53-63 (3073039)

65 52 and 64 (6530)

66 65 use emczd (5036)

67 34 or 66 (8720)

68 limit 67 to yr=“2011 -Current” (5297)

69 remove duplicates from 68 (4253)

70 67 not 68 (3423)

71 remove duplicates from 70 (2867)

72 69 or 71 (7120) [TOTAL UNIQUE RECORDS]

73 72 use prmz (3588) [UNIQUE MEDLINE RECORDS]

74 72 use emczd (3532) [UNIQUE EMBASE RECORDS]

Cochrane Library

Search Name: PRISMA - DTA - Reviews/Meta-Analyses - Methodology

Date Run: 05/05/16 14:41:54.305

Description: 2016 May 5 (OHRI)

ID Search Hits

#1 [mh ^“Diagnostic Techniques and Procedures”] 116

#2 [mh “Diagnostic Imaging”] 35671

#3 [mh “Diagnostic Tests, Routine”] 328

#4 (diagnos* near/3 test*):ti,ab,kw 6315

#5 (diagnos* near/3 accura*):ti,ab,kw 5863

#6 (test* near/3 accura*):ti,ab,kw 3703

#7 (diagnos* near/3 compar*):ti,ab,kw 1936

#8 (diagnostic next (study or studies)):ti,ab,kw 189

#9 DTA:ti,ab,kw 22

#10 (DTAR or DTARs):ti,ab,kw 2

#11 in Methods Studies 521

Methods – 521

## Appendix 2

**Table 2:** List of 203 included studies

1. A. Hoyer, O. Kuss. Meta-analysis of diagnostic tests accounting for disease prevalence: a new model using trivariate copulas. *Statistics in medicine*. 2015///. 34:1912
2. A.D. Kester, F. Buntinx. Meta-analysis of ROC curves. *Medical Decision Making*. 2000///. 20:430
3. A.H. Zwinderman, P.M. Bossuyt. We should not pool diagnostic likelihood ratios in systematic reviews. *Statistics in medicine*. 2008///. 27:687
4. A.K. Nikoloulopoulos. A mixed effect model for bivariate meta-analysis of diagnostic test accuracy studies using a copula representation of the random effects distribution. *Statistics in medicine*. 2015///. 34:3842
5. A.N.A. Tostesen, C.B. Begg. A general regression methodology for ROC curve estimation. *Medical Decision Making*. 1988///. 8:204
6. A.S. Midgette, T.A. Stukel, B. Littenberg. A meta-analytic method for summarizing diagnostic test performance: receiver-operating characteristic summary point estimates. *Medical Decision Making*. 1993///. 13:253
7. A.S. Rosman, M.A. Korsten. Application of summary receiver operating characteristic (sROC) analysis to diagnostic clinical testing. *Advances in medical sciences*. 2007///. 52:76
8. Alvaro Najib Atallah, Andrea Puchnick, Daniel Wu, David Carlos Shigueoka, Gianni Mara Silva dos Santos, Hernani Pinto de Jr. Lemos, Jose Eduardo Mourao, Wagner Iared. Remarks about systematic reviews of diagnostic tests. *Sao Paulo medical journal = Revista paulista de medicina*. 2012///. 130:279
9. Athina Tatsioni, Deborah A. Zarin, Naomi Aronson, David J. Samson, Carole R. Flamm, Christopher Schmid, Joseph Lau. Challenges in systematic reviews of diagnostic technologies. *Annals of internal medicine*. 2005///. 142:1048
10. B. Littenberg, L.E. Moses. Estimating diagnostic accuracy from multiple conflicting reports: a new meta-analytic method. *Medical decision making*: *an international journal of the Society for Medical Decision Making*. 1993///. 13:313
11. B. Opmeer, J. Reitsma, K. Broeze, B.W. Mol. The relation between heterogeneity in diagnostic accuracy, prevalence and patient characteristics: an illustration with individual patient data meta-analysis. Oral presentation at the 17th Cochrane Colloquium; 2009 Oct 11-14, Singapore [abstract]. *#journal#.* 2009///. Suppl:11
12. B.D. Thombs, E. Arthurs, G. El-Baalbaki, A. Meijer, R.C. Ziegelstein, R.J. Steele. Risk of bias from inclusion of patients who already have diagnosis of or are undergoing treatment for depression in diagnostic accuracy studies of screening tools for depression: systematic review. *BMJ* (*Online*). 2011///. 343:no
13. Behrouz Kassai, Sandrine Sonie, Nirav R. Shah, Jean Pierre Boissel. Literature search parameters marginally improved the pooled estimate accuracy for ultrasound in detecting deep venous thrombosis. *Journal of clinical epidemiology*. 2006///. 59:710
14. Ben A. Dwamena. Evidence-based radiology: step 3—diagnostic systematic review and meta-analysis (critical appraisal). *Seminars in roentgenology*. 2009///. 44:170
15. Bhurke S. Parekh, C.S. Kwok, C. Pang, L. Hooper, Y.K. Loke, J.J. Ryder, A.J. Sutton, C.B. Hing, I. Harvey, F. Song. Uptake of methods to deal with publication bias in systematic reviews has increased over time, but there is still much scope for improvement. *Journal of clinical epidemiology*. 2011///. 64:349
16. Brian H. Willis, Christopher J. Hyde. Estimating a test’s accuracy using tailored meta-analysis-how setting-specific data may aid study selection. *Journal of clinical epidemiology*. 2014///. 67:538
17. Brian H. Willis, Christopher J. Hyde. What is the test’s accuracy in my practice population? Tailored meta-analysis provides a plausible estimate. *Journal of clinical epidemiology*. 2015///. 68:847
18. Brian H. Willis, Muireann Quigley. The assessment of the quality of reporting of meta-analyses in diagnostic research: a systematic review. *BMC medical research methodology*. 2011///. 11:163
19. Brian H. Willis, Muireann Quigley. Uptake of newer methodological developments and the deployment of meta-analysis in diagnostic test research: a systematic review. *BMC medical research methodology*. 2011///. 11:27
20. Byron C. Wallace, Christopher H. Schmid, Joseph Lau, Thomas A. Trikalinos. Meta-analyst: software for meta-analysis of binary, continuous and diagnostic data. *BMC medical research methodology*. 2009///. 9:80
21. C. Davenport, C. Hyde. To what extent is the clinical context considered in diagnostic test accuracy reviews?: a methodological review. Oral presentation at the 19th Cochrane Colloquium; 2011 Oct 19-22; Madrid, Spain [abstract]. *#journal#.* 2011///. Suppl:9
22. C. Schmid, M. Chung, A. Tatsioni, L.L. Price, J. Lau. Evaluating heterogeneity in studies of diagnostic test accuracy [abstract]. *#journal#.* 2006///. #volume#:58
23. C. Schmid, M. Chung, P. Chew, J. Lau. Survey of diagnostic test meta-analyses [abstract]. *#journal#.* 2004///. #volume#:50
24. C.B. Begg. Meta-analysis methods for diagnostic accuracy. *Journal of clinical epidemiology*. 2008///. 61:1081
25. C.M. Jones, T. Athanasiou. Diagnostic accuracy meta-analysis: review of an important tool in radiological research and decision making. *The British journal of radiology*. 2009///. 82:441
26. C.M. Rutter, C.A. Gatsonis. A hierarchical regression approach to meta-analysis of diagnostic test accuracy evaluations. *Statistics in medicine*. 2001///. 20:2865
27. Christiana A. Naaktgeboren, Wynanda A. van Enst, Eleanor A. Ochodo, Joris A.H. de Groot, Lotty Hooft, Mariska M. Leeflang, Patrick M. Bossuyt, Karel G.M. Moons, Johannes B. Reitsma. Systematic overview finds variation in approaches to investigating and reporting on sources of heterogeneity in systematic reviews of diagnostic studies. *Journal of clinical epidemiology*. 2014///. 67:1200
28. Colin B. Begg. Systematic reviews of diagnostic accuracy studies require study by study examination: first for heterogeneity, and then for sources of heterogeneity. *Journal of clinical epidemiology*. 2005///. 58:865
29. Constantine Gatsonis, Prashni Paliwal. Meta-analysis of diagnostic and screening test accuracy evaluations: methodologic primer. *AJR.American journal of roentgenology*. 2006///. 187:271
30. D. Bohning, W. Bohning, H. Holling. Revisiting Youden’s index as a useful measure of the misclassification error in meta-analysis of diagnostic studies. *Statistical methods in medical research*. 2008///. 17:543
31. D. Stengel, K. Bauwens, J. Sehouli, A. Ekkernkamp, F. Porzsolt. A likelihood ratio approach to meta-analysis of diagnostic studies. *Journal of medical screening.* 2003///. 10:47
32. D.L. Simel, P.M. Bossuyt. Differences between univariate and bivariate models for summarizing diagnostic accuracy may not be large. *Journal of clinical epidemiology.* 2009///. 62:1292
33. D.S. MacDonald-Jankowski, M.F. Dozier. Systematic review in diagnostic radiology*. Dento maxillo facial radiology.* 2001///. 30:78
34. Danielle B. Rice, Ian Shrier, Lorie A. Kloda, Andrea Benedetti, Brett D. Thombs. Methodological quality of meta-analyses of the diagnostic accuracy of depression screening tools. *Journal of psychosomatic research*. 2016///. 84:84
35. Danlu Liu, Jiaxin Jin, Jinhui Tian, Kehu Yang. Quality assessment and factor analysis of systematic reviews and meta-analyses of endoscopic ultrasound diagnosis. *PloS one*. 2015///. 10:e0120911
36. Deville, L.M. Bouter, P.D. Bezemer, N. Yzermans, van der-Windt DAWM. Heterogeneity in systematic reviews of diagnostic studies [abstract]. *#journal#.* 1999///. #volume#:#pages#
37. E.C. Vamvakas. Meta-analyses of studies of the diagnostic accuracy of laboratory tests: a review of the concepts and methods. *Archives of pathology & laboratory medicine*. 1998///. 122:675
38. Eleanor A. Ochodo, Johannes B. Reitsma, Patrick M. Bossuyt, Mariska M.G. Leeflang. Survey revealed a lack of clarity about recommended methods for meta-analysis of diagnostic accuracy data. *Journal of clinical epidemiology*. 2013///. 66:1281
39. Eleanor A. Ochodo, Wynanda A. van Enst, Christiana A. Naaktgeboren, Joris A.H. de Groot, Lotty Hooft, Karel G.M. Moons, Johannes B. Reitsma, Patrick M. Bossuyt, Mariska M.G. Leeflang. Incorporating quality assessments of primary studies in the conclusions of diagnostic accuracy reviews: a cross-sectional study. *BMC medical research methodology*. 2014///. 14:33
40. Elodie Pambrun, Vincent Bouteloup, Rodolphe Thiebaut, Julien Asselineau, Victor de Ledinghen, Paul Perez, Steering Committee of the Transient Elastography Individual Patient Data meta-analysis Study (TE IPD Study). On the validity of meta-analyses: exhaustivity must be warranted, exclusion of duplicate patients too. *Journal of clinical epidemiology*. 2010///. 63:342
41. Erich P. Huang, Xiao Feng Wang, Kingshuk Roy Choudhury, Lisa M. McShane, Mithat Gonen, Jingjing Ye, Andrew J. Buckler, Paul E. Kinahan, Anthony P. Reeves, Edward F. Jackson, Alexander R. Guimaraes, Gudrun Zahlmann, Meta-Analysis Working Group. Meta-analysis of the technical performance of an imaging procedure: guidelines and statistical methodology. *Statistical methods in medical research*. 2015///. 24:141
42. F. Grossenbacher, M. Battaglia, A. Duss, D. Pewsner, H. Bucher, M. Egger. Searching for diagnostic text evaluations: the importance of specialist journals and databases. *#journal#.* 2002///. #volume#:#pages#
43. F.M. Chappell, G.M. Raab, J.M. Wardlaw. When are summary ROC curves appropriate for diagnostic meta-analyses? *Statistics in medicine*. 2009///. 28:2653
44. Francesco Sardanelli, Humayun Bashir, Dominik Berzaczy, Guglielmo Cannella, Ansgar Espeland, Nicola Flor, Thomas Helbich, Myriam Hunink, Dermot E. Malone, Ritse Mann, Claudia Muzzupappa, Lars J. Petersen, Katrine Riklund, Luca M. Sconfienza, Zbigniew Serafin, Sandra Spronk, Jaap Stoker, Edwin J.R. van Beek, Dierk Vorwerk, Giovanni Di Leo. The role of imaging specialists as authors of systematic reviews on diagnostic and interventional imaging and its impact on scientific quality: report from the EuroAIM Evidence-based Radiology Working Group. *Radiology*. 2014///. 272:533
45. Fujian Song, Khalid S. Khan, Jacqueline Dinnes, Alex J. Sutton. Asymmetric funnel plots and publication bias in meta-analyses of diagnostic accuracy. *International journal of epidemiology*. 2002///. 31:88
46. G. Ritchie, J. Glanville, C. Lefebvre. Do published search filters to identify diagnostic test accuracy studies perform adequately? *Health information and libraries journal*. 2007///. 24:188
47. Gary H. Lyman, Benjamin Djulbegovic. The challenge of systematic reviews of diagnostic and staging studies in cancer. *Cancer treatment reviews*. 2005///. 31:628
48. Geert Jan Geersing, Walter Bouwmeester, Peter Zuithoff, Rene Spijker, Mariska Leeflang, Karel G.M. Moons, Karel Moons. Search filters for finding prognostic and diagnostic prediction studies in Medline to enhance systematic reviews. *PloS one*. 2012///. 7:e32844
49. Georg M. Schuetz, Peter Schlattmann, Marc Dewey. Use of 3 × 2 tables with an intention to diagnose approach to assess clinical performance of diagnostic tests: meta-analytical evaluation of coronary CT angiography studies. *BMJ* (*Clinical research ed.*). 2012///. 345:e6717
50. Gerald W. Smetana, Craig A. Umscheid, Stephanie Chang, David B. Matchar. Methods guide for authors of systematic reviews of medical tests: a collaboration between the Agency for Healthcare Research and Quality (AHRQ) and the Journal of General Internal Medicine. *Journal of general internal medicine*. 2012///. 27 Suppl 1:S1
51. Gerta Rucker, Martin Schumacher. Summary ROC curve based on a weighted Youden index for selecting an optimal cutpoint in meta-analysis of diagnostic accuracy. *Statistics in medicine.* 2010///. 29:3069
52. H. Chu, L. Nie, S.R. Cole, C. Poole. Meta-analysis of diagnostic accuracy studies accounting for disease prevalence: alternative parameterizations and model selection. *Statistics in medicine*. 2009///. 28:2384
53. H. Chu, S.R. Cole. Bivariate meta-analysis of sensitivity and specificity with sparse data: a generalized linear mixed model approach. *Journal of clinical epidemiology*. 2006///. 59:1331
54. H. Putter, M. Fiocco, T. Stijnen. Meta-analysis of diagnostic test accuracy studies with multiple thresholds using survival methods. *Biometrical journal.Biometrische Zeitschrift*. 2010///. 52:95
55. H.C. de Vet, T. van der Weijden, J.W. Muris, J. Heyrman, F. Buntinx, J.A. Knottnerus. Systematic reviews of diagnostic research. Considerations about assessment and incorporation of methodological quality. *European journal of epidemiology*. 2001///. 17:301
56. H.C. Vet, T. Weijden, J.W. Muris, J. Heyrman, F. Buntinx, J.A. Knottnerus. Systematic reviews of diagnostic research considerations about assessment and incorporation of methodological quality. *European journal of epidemiology*. 2001///. 17:301
57. Haitao Chu, Hongfei Guo, Yijie Zhou. Bivariate random effects meta-analysis of diagnostic studies using generalized linear mixed models. *Medical decision making*: *an international journal of the Society for Medical Decision Making*. 2010///. 30:499
58. Haitao Chu, Hongfei Guo. A unification of models for meta-analysis of diagnostic accuracy studies. *Biostatistics* (*Oxford, England)*. 2009///. 10:201
59. Honest Honest, Khalid S. Khan. Reporting of measures of accuracy in systematic reviews of diagnostic literature. *BMC health services research.* 2002///. 2:4
60. I. Nicolau, D. Ling, L. Tian, C. Lienhardt, M. Pai. Quality and reporting of tuberculosis systematic reviews: Evaluation using amstar and prisma standard. *American journal of epidemiology*. 2011///. 173:S317
61. Issa J. Dahabreh, Mei Chung, Georgios D. Kitsios, Teruhiko Terasawa, Gowri Raman, Athina Tatsioni, Annette Tobar, Joseph Lau, Thomas A. Trikalinos, Christopher H. Schmid. Survey of the methods and reporting practices in published meta-analyses of test performance: 1987 to 2009. *Research synthesis methods*. 2013///. 4:242
62. J. Boissel, M. Cucherat. The meta-analysis of diagnostic test studies. *European radiology.* 1998///. 8:484
63. J. Burch, M. Westwood, Weiser K. Soares. Should data from case-controlled studies be included in systematic reviews alongside diagnostic cohort studies? [abstract]. *#journal#.* 2006///. #volume#:86
64. J. Cnossen, B.W. Mol, J. Post, Riet G. Ter. Is it necessary to perform full text papers selection by two independent reviewers in systematic reviews on diagnostic accuracy? [abstract]. *#journal#.* 2004///. #volume#:117
65. J. Deeks, A. Rutjes, J. Reitsma, M. Leeflang, P. Bossuyt. Statistical methods for investigating heterogeneity related to methodological quality in meta-analyses of studies of diagnostic accuracy [abstract]. *#journal#.* 2005///. #volume#:47
66. J. Deeks, P. Macaskill, L. Irwig. By how much does publication bias affect the results of systematic reviews of diagnostic test accuracy? [abstract]. *#journal#.* 2004///. #volume#:48
67. J. Deeks, P. Macaskill, L. Irwig. Detecting publication bias in systematic reviews of diagnostic test accuracy [abstract]. *#journal#.* 2004///. #volume#:47
68. J. Dinnes, J. Deeks, J. Kirby, P. Roderick. A methodological review of how heterogeneity has been examined in systematic reviews of diagnostic test accuracy. *Health technology assessment* (*Winchester, England*)*.* 2005///. 9:1
69. J. Doust, S. Sanders, P. Glasziou, E. Pietrzak. Identifying studies for systematic reviews of diagnostic tests [abstract]. *#journal#*. 2003///. #volume#:63
70. J. Dubourg, M. Berhouma, M. Cotton, M. Messerer. Meta-analysis of diagnostic test accuracy in neurosurgical practice. *Neurosurgical focus*. 2012///. 33:E5
71. J. Glanville, G. Ritchie, C. Lefebvre. How well do published search filters perform in finding diagnostic test accuracy studies? [abstract]. *#journal#*. 2008///. #volume#:18
72. J. Glanville, P. Whiting, J. Sterne, M. Westwood, M. Burke. Which databases should we search to identify diagnostic test accuracy studies? [abstract]. *#journal#*. 2007///. #volume#:294
73. J. Menke. Bivariate random-effects meta-analysis of sensitivity and specificity with SAS PROC GLIMMIX. *Methods of information in medicine.* 2010///. 49:54
74. J. Menten, M. Boelaert, E. Lesaffre. Bayesian meta-analysis of diagnostic tests allowing for imperfect reference standards. *Statistics in medicine.* 2013///. 32:5398
75. J.A. Doust, E. Pietrzak, S. Sanders, P.P. Glasziou. Identifying studies for systematic reviews of diagnostic tests was difficult due to the poor sensitivity and precision of methodologic filters and the lack of information in the abstract. *Journal of clinical epidemiology.* 2005///. 58:444
76. J.J. Deeks. Review on evidence-based cancer medicine—using evaluations of diagnostic tests: understanding their limitations and making the most of available evidence. *Annals of Oncology.* 1999///. 10:761
77. J.J. Deeks. Systematic reviews in health care: Systematic reviews of evaluations of diagnostic and screening tests. *British Medical Journal.* 2001///. 323:157
78. J.J. Deeks. Systematic reviews of evaluations of diagnostic and screening tests. *#journal#.* 2001///. #volume#:248
79. J.M. Glanville, M. Cikalo, F. Crawford, M. Dozier, P. Lowson. Handsearching for reports of diagnostic test accuracy studies: adding to the evidence base. Oral presentation at the Joint Cochrane and Campbell Colloquium; 2010 Oct 18-22; Keystone, Colorado, USA [abstract]. *#journal#.* 2010///. Suppl:48
80. James Hurley. Meta-analysis of clinical studies of diagnostic tests: developments in how the receiver operating characteristic “works”. *Archives of pathology & laboratory medicine.* 2011///. 135:1585
81. Jared M. Campbell, Miloslav Klugar, Sandrine Ding, Dennis P. Carmody, Sasja J. Hakonsen, Yuri T. Jadotte, Sarahlouise White, Zachary Munn. Diagnostic test accuracy: methods for systematic review and meta-analysis. *International journal of evidence-based healthcare.* 2015///. 13:154
82. Javier Zamora, Victor Abraira, Alfonso Muriel, Khalid Khan, Arri Coomarasamy. Meta-DiSc: a software for meta-analysis of test accuracy data. *BMC medical research methodology.* 2006///. 6:31
83. Jennifer Harbor, Cynthia Fraser, Carol Lefebvre, Julie Glanville, Sophie Beale, Charles Boachie, Steven Duffy, Rachael McCool, Lynne Smith. Reporting methodological search filter performance comparisons: a literature review. *Health information and libraries journal.* 2014///. 31:176
84. Jeroen G. Lijmer, Patrick M.M. Bossuyt, Siem H. Heisterkamp. Exploring sources of heterogeneity in systematic reviews of diagnostic tests. *Statistics in medicine.* 2002///. 21:1525
85. Jian Kang, Rollin Brant, William A. Ghali. Statistical methods for the meta-analysis of diagnostic tests must take into account the use of surrogate standards. *Journal of clinical epidemiology.* 2013///. 66:566
86. Johannes B. Reitsma, Afina S. Glas, Anne W.S. Rutjes, Rob J.P.M. Scholten, Patrick M. Bossuyt, Aeilko H. Zwinderman. Bivariate analysis of sensitivity and specificity produces informative summary measures in diagnostic reviews. *Journal of clinical epidemiology.* 2005///. 58:982
87. Johannes B. Reitsma, Karel G.M. Moons, Patrick M.M. Bossuyt, Kristian Linnet. Systematic reviews of studies quantifying the accuracy of diagnostic tests and markers. *Clinical chemistry.* 2012///. 58:1534
88. Jonathan J. Deeks, Petra Macaskill, Les Irwig. The performance of tests of publication bias and other sample size effects in systematic reviews of diagnostic test accuracy was assessed. *Journal of clinical epidemiology.* 2005///. 58:882
89. Joris Menten, Emmanuel Lesaffre. A general framework for comparative Bayesian meta-analysis of diagnostic studies. *BMC medical research methodology.* 2015///. 15:70
90. Juneyoung Lee, Kyung Won Kim, Sang Hyun Choi, Jimi Huh, Seong Ho Park. Systematic review and meta-analysis of studies Evaluating diagnostic test accuracy: a practical review for clinical researchers-part II. Statistical Methods of Meta-Analysis. *Korean journal of radiology.* 2015///. 16:1188
91. K. Bauwens, A. Ekkernkamp, D. Stengel. QUADAS: early experience with a new methodological scoring tool for diagnostic meta-analyses [abstract]. *#journal#.* 2005///. #volume#:74
92. K. Russell, N. Hooton, S. Blitz, C. Spooner, J. Beach, B. Rowe. Should sensitivities derived from 2 × 1 tables be included in diagnostic systematic reviews: a review of systematic reviews [abstract]. *#journal#.* 2005///. #volume#:125
93. K.E. Hartmann, D.B. Matchar, S. Chang. Chapter 6: Assessing applicability of medical test studies in systematic reviews. *Journal of general internal medicine.* 2012///. 27:S39
94. K.S. Khan, J. Dinnes, J. Kleijnen. Systematic reviews to evaluate diagnostic tests. *European Journal of Obstetrics Gynecology and Reproductive Biology.* 2001///. 95:6
95. K.S. Khan, L.M. Bachmann, Riet G. Ter. Systematic reviews with individual patient data meta-analysis to evaluate diagnostic tests. *European Journal of Obstetrics Gynecology and Reproductive Biology.* 2003///. 108:121
96. Khalid S. Khan. Systematic reviews of diagnostic tests: a guide to methods and application. *Best practice & research.Clinical obstetrics & gynecology.* 2005///. 19:37
97. L. Chong, R. Sun. Methodological developments and statistic software used in diagnostic systematic reviews in China. Poster presentation at the 19th Cochrane Colloquium; 2011 Oct 19-22; Madrid, Spain [abstract]. *#journal#.* 2011///. Suppl:144
98. L. Irwig, A.N. Tosteson, C. Gatsonis, J. Lau, G. Colditz, T.C. Chalmers, F. Mosteller. Guidelines for meta-analyses evaluating diagnostic tests. *Annals of internal medicine.* 1994///. 120:667
99. L. Irwig, P. Macaskill, P. Glasziou, M. Fahey. Meta-analytic methods for diagnostic test accuracy. *Journal of clinical epidemiology.* 1995///. 48:119
100. L. Manchikanti, R. Derby, L. Wolfer, V. Singh, S. Datta, J.A. Hirsch. Evidence-based medicine, systematic reviews, and guidelines in interventional pain management: part 7: systematic reviews and meta-analyses of diagnostic accuracy studies. *Pain physician.* 2009///. 12:929
101. L.E. Moses, D. Shapiro, B. Littenberg. Combining independent studies of a diagnostic test into a summary ROC curve: data-analytic approaches and some additional considerations. *Statistics in medicine.* 1993///. 12:1293
102. L.M. Bachmann, P. Estermann, C. Kronenberg, Riet G. Ter. Identifying diagnostic accuracy studies in EMBASE. *#journal#.* 2003///. 91:341
103. L.M. Bachmann, R. Coray, P. Estermann, Riet G. Ter. Identifying diagnostic studies in MEDLINE: reducing the number needed to read. *#journal#.* 2002///. 9:653
104. Long Ge, Jian Cheng Wang, Jin Long Li, Li Liang, Ni An, Xin Tong Shi, Yin Chun Liu, Jin Hui Tian. The assessment of the quality of reporting of systematic reviews/meta-analyses in diagnostic tests published by authors in China. *PloS one.* 2014///. 9:e85908
105. Louise Preston, Christopher Carroll, Paolo Gardois, Suzy Paisley, Eva Kaltenthaler. Improving search efficiency for systematic reviews of diagnostic test accuracy: an exploratory study to assess the viability of limiting to MEDLINE, EMBASE and reference checking. *Systematic reviews.* 2015///. 4:82
106. Lucy A. Parker, Noemi Gomez Saez, Miquel Porta, Ildefonso Hernandez-Aguado, Blanca Lumbreras. The impact of including different study designs in meta-analyses of diagnostic accuracy studies. *European journal of epidemiology.* 2013///. 28:713
107. Lukas P. Staub, Suzanne Dyer, Sarah J. Lord, R.John Simes. Linking the evidence: intermediate outcomes in medical test assessments. *International journal of technology assessment in health care.* 2012///. 28:52
108. M. Astin, M. Brazzelli, C. Fraser, C. Counsell, G. Needham, J. Grimshaw. A sensitive MEDLINE search strategy to retrieve studies of diagnostic imaging test performance [abstract]. *#journal#.* 2006///. #volume#:85
109. M. Brazzelli, P. Sandercock, *S. Lewis*, J. Deeks. Publication bias in studies of diagnostic accuracy in the stroke literature. What is the evidence? [abstract]. *#journal#.* 2008///. #volume#:21
110. M. Clarke. Systematic reviews: New challenges relating to non-randomised studies and diagnostic test accuracy. *Chinese Journal of Evidence-Based Medicine.* 2006///. 6:236
111. M. Hellmich, K.R. Abrams, A.J. Sutton. Bayesian approaches to meta-analysis of ROC curves. *Medical Decision Making.* 1999///. 19:252
112. M. Kastner, N.L. Wilczynski, A.K. McKibbon, A.X. Garg, R.B. Haynes. Diagnostic test systematic reviews: bibliographic search filters (“Clinical Queries”) for diagnostic accuracy studies perform well. *Journal of clinical epidemiology.* 2009///. 62:974
113. M. Lassere. Pooled metaanalysis of radiographic progression: comparison of Sharp and Larsen methods. *The Journal of rheumatology.* 2000///. 27:269
114. M. Leeflang, P.M. Bossuyt, L. Irwig. Sensitivity and specificity do vary with disease prevalence: implications for systematic reviews of diagnostic test accuracy [abstract]. *#journal#.* 2007///. #volume#:111
115. M. Leeflang, R. Scholten, H. Reitsma, A. Rutjes, J. Deeks, P. Bossuyt. Incorporating methodological quality in meta-analyses of diagnostic accuracy studies [abstract]. *#journal#.* 2005///. #volume#:81
116. M. Leeflang, R. Scholten, R. Reitsma, H. Rutjes, P. Bossuyt. Should diagnostic search filters be used in systematic reviews? [abstract]. *#journal#.* 2004///. #volume#:74
117. M. Pai, *L. Flores*, A. Hubbard, L. Riley, J. Colford. Quality assessment in meta-analyses of diagnostic studies: what difference does email contact with authors make? [abstract]. *#journal#.* 2003///. #volume#:36
118. M. Pai, M. McCulloch, W. Enanoria, Jr. Colford. Systematic reviews of diagnostic test evaluations: what’s behind the scenes?. *Evidence-based medicine.* 2004///. 9:101
119. M. Pennant, S. Wisniewski, C. Hyde, C. Davenport, J. Deeks. A tool to improve efficiency and quality in the production of protocols for Cochrane Reviews of Diagnostic Test Accuracy. Poster presentation at the 19th Cochrane Colloquium; 2011 Oct 19-22; Madrid, Spain [abstract]. *#journal#.* 2011///. Suppl:65
120. M. Wei, M. Liu. The current status of systematic reviews on diagnostic tests published in Chinese [abstract]. *#journal#.* 2007///. #volume#:170
121. M. Westwood, P. Whiting, J. Cooper, J. Kleijnen. Using the QUADAS tool in the conduct of systematic reviews of diagnostic tests [abstract]. *#journal#.* 2003///. #volume#:15
122. M. Westwood, P. Whiting. Should systematic reviews of diagnostic tests go beyond test accuracy? Poster presentation at the 16th Cochrane Colloquium: evidence in the era of globalisation; 2008 Oct 3-7; Freiburg, Germany [abstract]. *Zeitschrift fur Evidenz, Fortbildung und Qualitat im Gesundheitswesen.* 2008///. 102:66
123. M.D. Mitchell. Validation of the summary ROC for diagnostic test meta-analysis: a Monte Carlo simulation. *Academic radiology.* 2003///. 10:25
124. M.G. Myriam Hunink. Meta-analysis of diagnostic performance studies. *European journal of epidemiology.* 2013///. 28:711
125. M.M.G. Leeflang, R.J.P.M. Scholten, A.W.S. Rutjes, J.B. Reitsma, P.M.M. Bossuyt. Use of methodological search filters to identify diagnostic accuracy studies can lead to the omission of relevant studies. *Journal of clinical epidemiology.* 2006///. 59:234
126. M.M.G. Leeflang. Systematic reviews and meta-analyses of diagnostic test accuracy. *Clinical Microbiology and Infection.* 2014///. 20:105
127. M.P. Astin, M.G. Brazzelli, C.M. Fraser, C.E. Counsell, G. Needham, J.M. Grimshaw. Developing a sensitive search strategy in MEDLINE to retrieve studies on assessment of the diagnostic performance of imaging techniques. *Radiology.* 2008///. 247:365
128. M.R. De Sousa, A.L.P. Ribeiro. Systematic review and meta-analysis of diagnostic and prognostic studies: a tutorial. *Arquivos brasileiros de cardiologia.* 2009///. 92:235
129. M.S. Siadaty, J. Shu. Proportional odds ratio model for comparison of diagnostic tests in meta-analysis. *BMC medical research methodology.* 2004///. 4:27
130. Marcos R. Sousa, Antonio Luiz Ribeiro. Systematic review and meta-analysis of diagnostic and prognostic studies: a tutorial. *Arquivos brasileiros de cardiologia.* 2009///. 92:229
131. Marie E. Westwood, Penny F. Whiting, Jos Kleijnen. How does study quality affect the results of a diagnostic meta-analysis?. *BMC medical research methodology.* 2005///. 5:20
132. Mariska Leeflang, Johannes Reitsma, Rob Scholten, Anne Rutjes, Marcello Di Nisio, Jon Deeks, Patrick Bossuyt. Impact of adjustment for quality on results of metaanalyses of diagnostic accuracy. *Clinical chemistry.* 2007///. 53:164
133. Mariska M.G. Leeflang, Jonathan J. Deeks, Constantine Gatsonis, Patrick M.M. Bossuyt, Cochrane Diagnostic Test Accuracy Working Group. Systematic reviews of diagnostic test accuracy. *Annals of internal medicine.* 2008///. 149:889
134. Mariska M.G. Leeflang, Jonathan J. Deeks, Yemisi Takwoingi, Petra Macaskill. Cochrane diagnostic test accuracy reviews. *Systematic reviews.* 2013///. 2:82
135. Matthew D.F. McInnes, Patrick M.M. Bossuyt. Pitfalls of systematic reviews and meta-analyses in imaging research. *Radiology.* 2015///. 277:13
136. N. Novielli, N.J. Cooper, A.J. Sutton, K.R. Abrams. Which meta-analysis model best fits my diagnostic test data? Use of model fit statistics. Oral presentation at the 16th Cochrane Colloquium: Evidence in the era of globalization; 2008 Oct 3-7; Freiburg, Germany [abstract]. *Zeitschrift fur Evidenz, Fortbildung und Qualitat im Gesundheitswesen.* 2008///. 102:10
137. N.P. Johnson, K.S. Khan. Gynaecologists blaze the trail in primary studies and systematic reviews of diagnostic test accuracy. *Australian and New Zealand Journal of Obstetrics and Gynaecology.* 2009///. 49:71
138. P. Macaskill. Empirical Bayes estimates generated in a hierarchical summary ROC analysis agreed closely with those of a full Bayesian analysis. *Journal of clinical epidemiology.* 2004///. 57:925
139. P. Michel, E. Mouillet, L.R. Salmi. Comparison of Medical Subject Headings and standard terminology regarding performance of diagnostic tests. *#journal#.* 2006///. 94:221
140. P. Whiting, A. Rutjes, J. Reitsma, P. Bossuyt, J. Kleijnen. QUADAS: the development of a new tool for the quality assessment of diagnostic accuracy studies [abstract]. *#journal#.* 2003///. #volume#:38
141. P. Whiting, A. Rutjes, M. Westwood, S. Mallett, J. Deeks, J. Reitsma, M. Leeflang, J. Sterne, P. Bossuyt. QUADAS-2: an updated quality assessment tool for diagnostic accuracy studies. Oral presentation at the 19th Cochrane Colloquium: scientific evidence for healthcare quality and patient safety; 2011 Oct 19-22; Madrid, Spain [abstract]. *#journal#.* 2011///. Suppl:14
142. P. Whiting, M. Westwood, J. Deeks, R. Harbord, L. Bachmann, M. Egger, J. Sterne. Graphical presentation of diagnostic information: a methodological review [abstract]. *#journal#.* 2005///. #volume#:109
143. P. Whiting, M. Westwood, M. Burke, J. Sterne, J. Glanville. Is it necessary to search a wide range of databases to identify diagnostic test accuracy studies? [abstract]. *#journal#.* 2006///. #volume#:89
144. P. Whiting, R. Gupta, J. Burch, J. Kleijnen, A. Marson, C. Forbes. What to do with non 2 × 2 data from a diagnostic systematic review? An example from a review on identifying the seizure focus in patients with epilepsy [abstract]. *#journal#.* 2004///. #volume#:203
145. P.E. Verde. Meta-analysis of diagnostic test data: a bivariate Bayesian modeling approach. *Statistics in medicine.* 2010///. 29:3088
146. P.F. Whiting, J.A. Sterne, M.E. Westwood, L.M. Bachmann, R. Harbord, M. Egger, J.J. Deeks. Graphical presentation of diagnostic information. *BMC medical research methodology.* 2008///. 8:20
147. P.F. Whiting, M.E. Westwood, M. Burke, J.A.C. Sterne, J. Glanville. Can diagnostic filters offer similar sensitivity and a reduced number needed to read compared to searches based on index test and target condition? Oral presentation at the 16th Cochrane Colloquium: Evidence in the era of globalization; 2008 Oct 3-7; Freiburg, Germany [abstract]. *Zeitschrift fur Evidenz, Fortbildung und Qualitat im Gesundheitswesen.* 2008///. 102:10
148. P.Lina Santaguida, Crystal M. Riley, David B. Matchar. Chapter 5: assessing risk of bias as a domain of quality in medical test studies. *Journal of general internal medicine.* 2012///. 27 Suppl 1:S33
149. P.M.M. Bossuyt. Informative reporting of systematic reviews in radiology. *Radiology.* 2013///. 269:313
150. Paul Christian Burkner, Philipp Doebler. Testing for publication bias in diagnostic meta-analysis: a simulation study. *Statistics in medicine.* 2014///. 33:3061
151. Paul Cronin, James V. Rawson. Review of research reporting guidelines for radiology researchers. *Academic radiology.* 2016///. 23:537
152. Penny F. Whiting, Anne W.S. Rutjes, Marie E. Westwood, Susan Mallett, Jonathan J. Deeks, Johannes B. Reitsma, Mariska M.G. Leeflang, Jonathan A.C. Sterne, Patrick M.M. Bossuyt, QUADAS-2 Group. QUADAS-2: a revised tool for the quality assessment of diagnostic accuracy studies. *Annals of internal medicine.* 2011///. 155:529
153. Penny F. Whiting, Marie E. Weswood, Anne W.S. Rutjes, Johannes B. Reitsma, Patrick N.M. Bossuyt, Jos Kleijnen. Evaluation of QUADAS, a tool for the quality assessment of diagnostic accuracy studies. *BMC medical research methodology.* 2006///. 6:9
154. Penny Whiting, Anne W.S. Rutjes, Jacqueline Dinnes, Johannes B. Reitsma, Patrick M.M. Bossuyt, Jos Kleijnen. A systematic review finds that diagnostic reviews fail to incorporate quality despite available tools. *Journal of clinical epidemiology.* 2005///. 58:1
155. Penny Whiting, Anne W.S. Rutjes, Johannes B. Reitsma, Patrick M.M. Bossuyt, Jos Kleijnen. The development of QUADAS: a tool for the quality assessment of studies of diagnostic accuracy included in systematic reviews. *BMC medical research methodology.* 2003///. 3:25
156. Penny Whiting, Marie Westwood, Margaret Burke, Jonathan Sterne, Julie Glanville. Systematic reviews of test accuracy should search a range of databases to identify primary studies. *Journal of clinical epidemiology.* 2008///. 61:357
157. Penny Whiting, Marie Westwood, Rebecca Beynon, Margaret Burke, Jonathan Ac Sterne, Julie Glanville. Inclusion of methodological filters in searches for diagnostic test accuracy studies misses relevant studies. *Journal of clinical epidemiology.* 2011///. 64:602
158. Penny Whiting, Roger Harbord, Jos Kleijnen. No role for quality scores in systematic reviews of diagnostic accuracy studies. *BMC medical research methodology.* 2005///. 5:19
159. Peter Schlattmann, Maryna Verba, Marc Dewey, Mario Walther. Mixture models in diagnostic meta-analyses—clustering summary receiver operating characteristic curves accounted for heterogeneity and correlation. *Journal of clinical epidemiology.* 2015///. 68:61
160. R. Beynon, M. Leeflang, A. Eisinga, S. McDonald, R. Mitchell. A systematic review of studies that develop or evaluate search filters for the retrieval of diagnostic studies in MEDLINE. Oral presentation at the 19th Cochrane Colloquium; 2011 Oct 19-22; Madrid, Spain [abstract]. *#journal#.* 2011///. Suppl:20
161. R. Harbord, L. Bachmann, A. Shang, P. Whiting, J. Deeks, M. Egger, J. Sterne. An empirical comparison of methods for meta-analysis of studies of diagnostic accuracy [abstract]. *#journal#.* 2005///. #volume#:46
162. R. Harbord, L. Bachmann, J. Deeks, P. Whiting, M. Egger, J. Sterne. Meta-analysis of studies of diagnostic accuracy: a unified approach [abstract]. *#journal#.* 2005///. #volume#:45
163. R.D. Riley, S.R. Dodd, J.V. Craig, J.R. Thompson, P.R. Williamson. Meta-analysis of diagnostic test studies using individual patient data and aggregate data. *Statistics in medicine.* 2008///. 27:6111
164. Richard D. Riley, Ikhlaaq Ahmed, Joie Ensor, Yemisi Takwoingi, Amanda Kirkham, R.Katie Morris, J.Pieter Noordzij, Jonathan J. Deeks. Meta-analysis of test accuracy studies: an exploratory method for investigating the impact of missing thresholds. *Systematic reviews.* 2015///. 4:12
165. R.D. Riley, S.R. Dodd, J.V. Craig, P.R. Williamson. Meta-analysis of diagnostic test studies using individual patient data and aggregate data. Oral presentation at the 16th Cochrane Colloquium: evidence in the era of globalisation; 2008 Oct 3-7; Freiburg, Germany [abstract]. *Zeitschrift fur Evidenz, Fortbildung und Qualitat im Gesundheitswesen.* 2008///. 102:28
166. R.M. Harbord, J.J. Deeks, M. Egger, P. Whiting, J.A. Sterne. A unification of models for meta-analysis of diagnostic accuracy studies. *Biostatistics* (*Oxford, England*)*.* 2007///. 8:239
167. Rachel Mann, Simon M. Gilbody. Should methodological filters for diagnostic test accuracy studies be used in systematic reviews of psychometric instruments? A case study involving screening for postnatal depression. *Systematic reviews.* 2012///. 1:9
168. Rebecca Beynon, Mariska M.G. Leeflang, Steve McDonald, Anne Eisinga, Ruth L. Mitchell, Penny Whiting, Julie M. Glanville. Search strategies to identify diagnostic accuracy studies in MEDLINE and EMBASE. *The Cochrane database of systematic reviews.* 2013///. 9:MR000022
169. Richard D. Riley, Ikhlaaq Ahmed, Thomas P.A. Debray, Brian H. Willis, J.Pieter Noordzij, Julian P.T. Higgins, Jonathan J. Deeks. Summarising and validating test accuracy results across multiple studies for use in clinical practice. *Statistics in medicine.* 2015///. 34:2081
170. Roger M. Harbord, Penny Whiting, Jonathan A.C. Sterne, Matthias Egger, Jonathan J. Deeks, Aijing Shang, Lucas M. Bachmann. An empirical comparison of methods for meta-analysis of diagnostic accuracy showed hierarchical models are necessary. *Journal of clinical epidemiology.* 2008///. 61:1095
171. S. Halligan. Systematic reviews and meta-analysis of diagnostic tests. *Clinical radiology.* 2005///. 60:977
172. S. Mallett, J. Deeks, D. Altman. Treatment of heterogeneity in systematic reviews of diagnostic tests in cancer [abstract]. *#journal#.* 2004///. #volume#:159
173. S. Mallett, N. Summerton, J. Deeks, S. Halligan, D. Altman. Systematic reviews of diagnostic tests in cancer: assessment of methodology and reporting quality [abstract]. *#journal#.* 2003///. #volume#:15
174. S. Sandberg. Systematic reviews of diagnostic tests—a new challenge for laboratory medicine. *Scandinavian Journal of Clinical and Laboratory Investigation.* 1997///. 57:369
175. S.D. Walter, L. Irwig, P.P. Glasziou. Meta-analysis of diagnostic tests with imperfect reference standards. *Journal of clinical epidemiology.* 1999///. 52:943
176. S.D. Walter, T. Sinuff. Studies reporting ROC curves of diagnostic and prediction data can be incorporated into meta-analyses using corresponding odds ratios. *Journal of clinical epidemiology.* 2007///. 60:530
177. Sadao Suzuki, Takeo Moro-oka, Niteesh K. Choudhry. The conditional relative odds ratio provided less biased results for comparing diagnostic test accuracy in meta-analyses. *Journal of clinical epidemiology.* 2004///. 57:461
178. Sheetal Parekh-Bhurke, Chun S. Kwok, Chun Pang, Lee Hooper, Yoon K. Loke, Jon J. Ryder, Alex J. Sutton, Caroline B. Hing, Ian Harvey, Fujian Song. Uptake of methods to deal with publication bias in systematic reviews has increased over time, but there is still much scope for improvement. *Journal of clinical epidemiology.* 2011///. 64:349
179. Shelley S. Selph, Alexander D. Ginsburg, Roger Chou. Impact of contacting study authors to obtain additional data for systematic reviews: diagnostic accuracy studies for hepatic fibrosis. *Systematic reviews.* 2014///. 3:107
180. Steve Halligan, Douglas G. Altman. Evidence-based practice in radiology: steps 3 and 4—appraise and apply systematic reviews and meta-analyses. *Radiology.* 2007///. 243:13
181. Susan Mallett, Jonathan J. Deeks, Steve Halligan, Sally Hopewell, Victoria Cornelius, Douglas G. Altman. Systematic reviews of diagnostic tests in cancer: review of methods and reporting. *BMJ* (*Clinical research ed.*). 2006///. 333:413
182. T.H. Hamza, H.C. Houwelingen, M.H. Heijenbrok-Kal, T. Stijnen. Associating explanatory variables with summary receiver operating characteristic curves in diagnostic meta-analysis. *Journal of clinical epidemiology.* 2009///. 62:1284
183. T.H. Hamza, J.B. Reitsma, T. Stijnen. Meta-analysis of diagnostic studies: a comparison of random intercept, normal-normal, and binomial-normal bivariate summary ROC approaches. *Medical Decision Making.* 2008///. 28:639
184. T.H. Hamza, L.R. Arends, H.C. Houwelingen, T. Stijnen. Multivariate random effects meta-analysis of diagnostic tests with multiple thresholds. *BMC medical research methodology.* 2009///. 9:73
185. Taye H. Hamza, Hans C. van Houwelingen, Theo Stijnen. The binomial distribution of meta-analysis was preferred to model within-study variability. *Journal of clinical epidemiology.* 2008///. 61:41
186. Thomas A. Trikalinos, David C. Hoaglin, Kevin M. Small, Norma Terrin, Christopher H. Schmid. Methods for the joint meta-analysis of multiple tests. *Research synthesis methods.* 2014///. 5:294
187. V. Dukic, C. Gatsonis. Meta-analysis of diagnostic test accuracy assessment studies with varying number of thresholds. *Biometrics.* 2003///. 59:936
188. V. Hasselblad, L.V. Hedges. Meta-analysis of screening and diagnostic tests. *Psychological bulletin.* 1995///. 117:167
189. V. Velanovich. Meta-analysis for combining Bayesian probabilities. *#journal#.* 1991///. 35:192
190. W. Devill. Meta-analysis of diagnostic research: does an optimal weight factor exists?. *#journal#.* 2000///. #volume#:34
191. W. Deville, N. Yzermans, L.M. Bouter, P.D. Bezemer, D.A.W. Windt. Heterogeneity in systematic reviews of diagnostic studies [abstract]. *#journal#.* 1999///. #volume#:#pages#
192. W.A. Ernst, R.J.P.M. Scholten, L. Hooft. Could a search for a diagnostic test accuracy review be restricted to MEDLINE? Oral presentation at the 19th Cochrane Colloquium; 2011 Oct 19-22; Madrid, Spain [abstract]. *#journal#.* 2011///. Suppl:19
193. W.Annefloor van Enst, Eleanor Ochodo, Rob J.P.M. Scholten, Lotty Hooft, Mariska M. Leeflang. Investigation of publication bias in meta-analyses of diagnostic test accuracy: a meta-epidemiological study. *BMC medical research methodology.* 2014///. 14:70
194. W.L. Devill. Pooling diagnostic publications: watch for outliers! [abstract]. *#journal#.* 1997///. #volume#:280
195. W.L. Deville, F. Buntinx. Guidelines for conducting systematic reviews of studies evaluating the accuracy of diagnostic tests. *#journal#.* 2002///. #volume#:145
196. W.P. Oosterhuis, R.W. Niessen, P.M. Bossuyt. The science of systematic reviewing studies of diagnostic tests. *Clinical chemistry and laboratory medicine.* 2000///. 38:577
197. Walter L. Deville, Frank Buntinx, Lex M. Bouter, Victor M. Montori, Henrica C.W. de Vet, Danielle A.W.M. van der Windt, P.Dick Bezemer. Conducting systematic reviews of diagnostic studies: didactic guidelines. *BMC medical research methodology.* 2002///. 2:9
198. William Hollingworth, L.Santiago Medina, Robert E. Lenkinski, Dean K. Shibata, Byron Bernal, David Zurakowski, Bryan Comstock, Jeffrey G. Jarvik. Interrater reliability in assessing quality of diagnostic accuracy studies using the QUADAS tool. A preliminary assessment. *Academic radiology.* 2006///. 13:803
199. Wynanda Annefloor van Enst, Christiana A. Naaktgeboren, Eleanor A. Ochodo, Joris A.H. de Groot, Mariska M. Leeflang, Johannes B. Reitsma, Rob J.P.M. Scholten, Karel G.M. Moons, Aeilko H. Zwinderman, Patrick M.M. Bossuyt, Lotty Hooft. Small-study effects and time trends in diagnostic test accuracy meta-analyses: a meta-epidemiological study. *Systematic reviews.* 2015///. 4:66
200. Yemisi Takwoingi, Mariska M.G. Leeflang, Jonathan J. Deeks. Empirical evidence of the importance of comparative studies of diagnostic test accuracy. *Annals of internal medicine.* 2013///. 158:544
201. Yemisi Takwoingi, Richard D. Riley, Jonathan J. Deeks. Meta-analysis of diagnostic accuracy studies in mental health. *Evidence-based mental health.* 2015///. 18:103
202. Z. Zhelev, R. Garside, C. Hyde. Investigating and improving the understanding of Cochrane diagnostic test accuracy reviews. Oral presentation at the 19th Cochrane Colloquium; 2011 Oct 19-22; Madrid, Spain [abstract]. *#journal#.* 2011///. Suppl:8
203. Zhivko Zhelev, Ruth Garside, Christopher Hyde. A qualitative study into the difficulties experienced by healthcare decision makers when reading a Cochrane diagnostic test accuracy review. *Systematic reviews.* 2013///. 2:32

## Abbreviations

AHRQ: Agency for Healthcare Research and Quality
AMSTAR: A Measurement Tool to Assess Systematic reviews
DTA: Diagnostic test accuracy
EQUATOR: Enhancing the QUAlity and Transparency Of health Research
MECIR: Methodological Expectations of Cochrane Intervention Reviews
MOOSE: Meta-analysis of Observational Studies in Epidemiology
PRESS: Peer Review of Electronic Search Strategies
PRISMA: Preferred Reporting Items for Systematic Reviews and Meta-Analyses
PRISMA-DTA: Preferred Reporting Items for Systematic Reviews and Meta-Analyses of Diagnostic Test Accuracy
QUADAS: QUality Assessment of Diagnostic Accuracy Studies
ROBIS: Risk of Bias in Systematic Reviews
STARD: Standards for Reporting Diagnostic Accuracy
